# Discoidin Domain Receptor-1 (DDR1) is Involved in Angiolymphatic Invasion in Oral Cancer

**DOI:** 10.3390/cancers12040841

**Published:** 2020-03-31

**Authors:** Yu-Lian Chen, Wan-Hua Tsai, Ying-Chieh Ko, Ting-Yu Lai, Ann-Joy Cheng, Shine-Gwo Shiah, Jenn-Ren Hsiao, Jang-Yang Chang, Su-Fang Lin

**Affiliations:** 1 National Institute of Cancer Research, National Health Research Institutes, Miaoli County 35053, Taiwan; 2 Institute of Bioinformatics and Structural Biology, National Tsing-Hua University, Hsinchu 30013, Taiwan; 3 Department of Medical Biotechnology, Chang Gung University, Taoyuan 33302, Taiwan; 4 Department of Otolaryngology, Head and Neck Collaborative Oncology Group, National Cheng Kung University Hospital, College of Medicine, National Cheng Kung University, Tainan 70403, Taiwan; 5 Department of Internal Medicine, National Cheng Kung University Hospital, College of Medicine, National Cheng Kung University, Tainan 70403, Taiwan

**Keywords:** oral squamous cell carcinoma (OSCC), discoidin domain receptor-1 (DDR1), angiolymphatic invasion (ALI), collective cancer cell migration

## Abstract

The discoidin domain receptor-1 (DDR1) is a non-integrin collagen receptor recently implicated in the collective cell migration of other cancer types. Previously, we identified an elevated expression of DDR1 in oral squamous cell carcinoma (OSCC) cells. Through the data mining of a microarray dataset composed of matched tumor-normal tissues from forty OSCC patients, we distilled overexpressed genes statistically associated with angiolymphatic invasion, including DDR1, COL4A5, COL4A6 and PDPN. Dual immunohistochemical staining further confirmed the spatial locations of DDR1 and PDPN in OSCC tissues indicative of collective cancer cell invasion. An elevated DDR1 expression at both the transcription and protein level was observed by treating keratinocytes with collagen of fibrillar or basement membrane types. In addition, inhibition of DDR1 kinase activity in OSCC TW2.6 cells disrupted cell cohesiveness in a 2D culture, reduced spheroid invasion in a collagen gel matrix, and suppressed angiolymphatic invasion in xenograft tissues. Taken together, these results suggest that collagen deposition in the affected tissues followed by DDR1 overexpression could be central to OSCC tumor growth and angiolymphatic invasion. Thus, DDR1 inhibitors are potential therapeutic compounds in restraining oral cancer, which has not been previously explored.

## 1. Introduction

Head and neck cancer is the sixth most common cancer worldwide; tumors from the oral cavity constitute the most common type of tumor in head and neck cancer [1]. In Taiwan, owing to betel quid (BQ) chewing habits, oral cancer incidence and mortality rate has risen rapidly, which prompted many national acts for preventing this disease, including screening patients whose oral lesions are still at premalignant stages. According to the latest report of the Taiwan Cancer Registry Database, the five-year overall survival rate of oral cancer is 55.88% [2]. 

Similar to other epithelial carcinomas, oral cancer represents the final outcome of a multi-year progression from benign hyperplasia to dysplasia, and from carcinoma in situ to invasive cancer [3]. The use of BQ itself is regarded as carcinogenic to humans [4]. Indeed, BQ with or without tobacco is closely associated with various forms of oral premalignant lesions and oral cancer [5]. BQ ingredients are known to provoke tissue inflammation while weakening the local immune surveillance system [6]. In addition, biologically active alkaloids, polyphenols, and trace elements of BQ aggressively alter the integrity of the associated oral epithelium, including the accumulation of collagen in the affected tissue [7]. Emerging evidence also indicates that, in BQ chewers, TGFB1-collagen deposition plays an important role in oral cancer progression [8,9,10,11].

The discoidin domain receptor-1 (DDR1) acquired its name from sharing extracellular domains with the discoidin receptors of *Dictyostelium discoideum*, a social slime mold that dynamically changes its shape between single amoeba and multicellular slug under starvation. Interestingly, prior results also demonstrated that DDR1 is involved in cell spreading as well as aggregation via regulation of cell surface CDH1 stability [12,13]. Molecularly, DDR1 is a non-integrin-type receptor for collagens and is composed of an N-terminal ligand binding ectodomain, a long juxtamembrane domain intercepted by a transmembrane motif, and a C-terminal intracellular kinase domain [14]. Upon activation, DDR1 undergoes autophosphorylation at multiple tyrosine residues located at the intracellular regions. Phosphorylation of DDR1 recruits cytoplasmic signaling adaptors containing Src homology-2 (SH2) or phosphotyrosine-binding (PTB) motifs, which in turn assemble more signaling molecules to execute various biological processes involved in cell migration, differentiation, and extracellular matrix (ECM) remodeling [15,16]. Overexpression of DDR1 has been detected in a variety of tumor types and plays diverse oncogenic roles, including prosurvival cell growth and chemoresistance (reviewed in [14]). In addition, Hidalgo-Carcedo et al. recognized that DDR1 also has a role in the collective cell invasion of cutaneous squamous cell carcinoma and breast cancer [17].

Collective cell invasion, defined as a multicellular movement with polarity, is a common pathological feature in many cancer types including oral squamous cell carcinoma (OSCC) [18,19]. Mechanistically, collective cell migration requires an array of basal epithelium molecules, adherent junctions and cadherins to maintain integrity and cell-cell coordination during collective movement [20,21,22]. At molecular level, DDR1, PARD3, PARD6, CDH1, and CLDN11 are responsible for maintaining cell–cell junctions during cluster advancement [17,23]; PDPN, a mucin-like transmembrane protein frequently expressed in the leading edge of tumor, is implicated in guiding movement direction [24,25,26]; matrix metalloproteinases (MMPs) and basement membrane type IV collagens are required for track clearing and secondary ECM remodeling, respectively [27]. Based on histological evidence, collective cancer cell migration is likely to be a critical step in cancer metastasis [28].

Previously, we showed that DDR1 is one of the top five protein tyrosine kinases overexpressed in BQ-associated OSCC cell lines [29]; overexpression of DDR1 in OSCC tissues is statistically associated with three clinical features: angiolymphatic invasion (ALI), perineural invasion (PNI), and lymph node metastasis [30]. In the present study, we validated DDR1-associated collective cell invasion in OSCC tumor sections, and provided evidence that the small molecule inhibitor DDR1-IN-1 suppressed ALI in a mouse model with statistical significance.

## 2. Results

### 2.1. Clinical Relevance of DDR1 Overexpression and ALI

**Figure 1:**
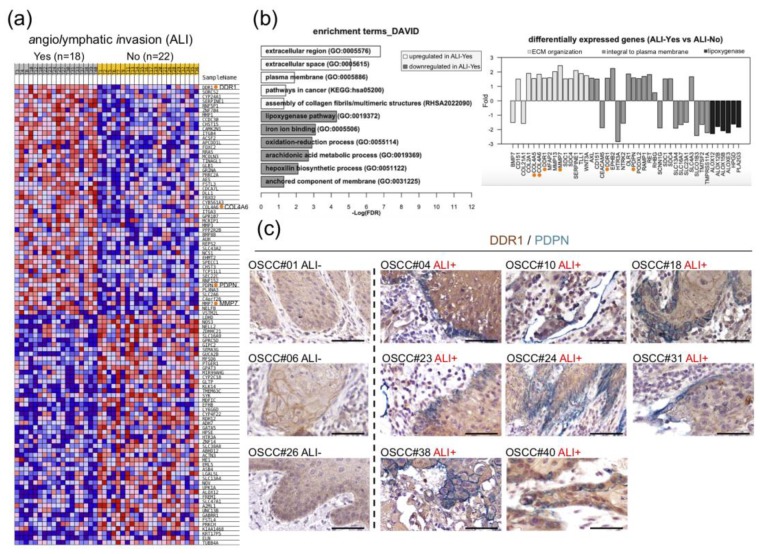
Upregulation of DDR1 and co-localization of DDR1 and PDPN in the invasion front of OSCC tissues with angiolymphatic invasion (ALI). (**a**) Heat map shows top 100 genes differentially expressed in ALI-Yes (*n* = 18) and ALI-No (*n* = 22). Expression values of high, moderate, low, and lowest are represented as red, pink, light blue, and dark blue, respectively [32]. DDR1, COL4A6, PDPN, and MMP7 are marked with orange dots. (**b**) Enriched gene ontology terms (left panel, FDR < 0.05) and selected differentially expressed genes (right panel, fold change > 1.5) in ALI. COL4A5, COL4A6, DDR1, MMP7 and PDPN are denoted with orange dots. (**c**) Representative immunohistochemistry images of OSCC tissues stained for DDR1 (brown) and PDPN (green). Scale bars, 50 μm.

**Figure A1:**
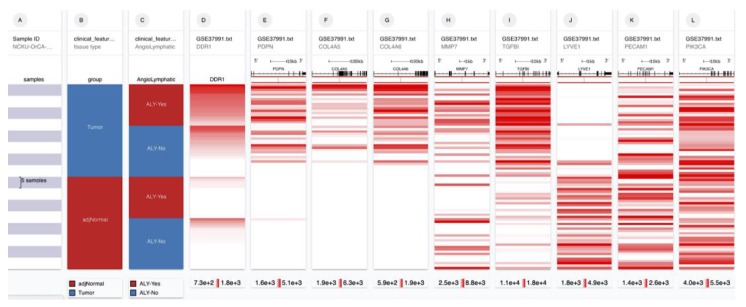
Integrated associations of angiolymphatic invasion phenotype (column C) of NCKU-OrCA-40TN (column B: 40T in blue; 40N in red), with gene expression of DDR1, PDPN, COL4A5, COL4A6, MMP7, and TGFBI by using UCSC Xena platform [59]. LYVE1 (lymphatic vessel marker), PECAM1 (CD31, vascular vessel marker), and PIK3CA are included as controls.

Dataset GSE37991 is a microarray-based transcriptome profile of 40 matched pairs of BQ-associated OSCC and adjacent normal tissues, dubbed NCKU-OrCA-40TN, established previously [31]. In this cohort, ALI and PNI, two pathological features that might involve collective cell migration, were noted in 18 (45%) and 25 (62.5%) of the 40 tumor tissues, respectively. Differential expression analysis using *GSEA* [32] and *Limma* [33] of the 40 tumor transcriptomes consistently revealed DDR1, PDPN, and COL4A6 as top upregulated genes in tissues with ALI compared to those without (Figure 1a, Table A1, Figure A1). In addition, functional annotation by using *DAVID* [34] showed that upregulated genes in ALI samples are statistically clustered in ‘extracellular region and plasma membrane’, ‘pathways in cancer’, and ‘assembly of collagen fibrils or multimeric structures’. On the other hand, downregulated genes are enriched in multiple cellular lipid metabolic processes and ‘iron ion binding’ (Figure 1b, left). Individually, upregulation of DDR1, COL4A5, COL4A6 and MMP7 contribute to ‘ECM organization’; DDR1 and PDPN are ‘integral plasma membrane’ molecules (Figure 1b, right). To validate overexpression of DDR1 and PDPN are associated with ALI in OSCC tissues, dual immunohistochemical staining was conducted in available FFPE tissues, including eight samples with ALI and three without. As shown in Figure 1c and Figure S1, DDR1 staining is more evident in samples with ALI than those without, whereas PDPN expression is uniquely localized in the invasion front or outward regions of ALI positive tumor sections. Note that the PDPN staining is reminiscent of poor prognosis markers previously documented in oral cancer and premalignant lesions [24,26,35].

### 2.2. Expression Levels of DDR1 and Basement Membrane Type IV Collagen are Positively Correlated

**Figure 2:**
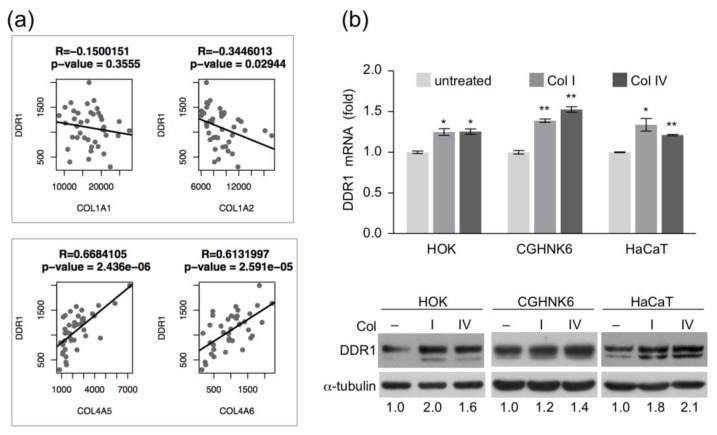
Correlation of collagen and DDR1 expression in vivo and in vitro. (**a**) Pearson correlation analysis between the expression levels of DDR1 and that of collagen type I (upper panel) and type IV (lower panel) in the 40 tumor tissues of NCKU-OrCA-40TN. DDR1 is positively correlated with COL4A5 and –A6, but not COL1A1 or –A2. (**b**) (upper) RT-qPCR assay of DDR1 mRNA levels in human keratinocytes treated with collagen (Col I or Col IV, 10 μg/mL) for 12 h. Data are presented as mean ± SD from triplicate assays. Statistical evaluation was performed with Student’s *t*-test. * *p* < 0.05; ** *p* < 0.01. Similar results were obtained from two experiments; one set of data is shown (lower). Western blot analysis of DDR1 protein levels in human keratinocytes treated with type I or type IV collagen for 24 h α-tubulin served as an internal control.

Because DDR1 is a collagen receptor and collagen accumulation in the oral cavity is an early event in OSCC pathogenesis, we asked whether DDR1 overexpression is concurrent with collagen accumulation in the 40 tumor tissues of NCKU-OrCA-40TN. To this end, the gene expression levels between DDR1 and eight collagen subunits in the dataset, including type I fibrillar (COL1A1, –A2) and type IV basement membrane (COL4A1, –A2, –A3, –A4, –A5, –A6), were individually evaluated by using Pearson correlation coefficient test. The results showed that expression of DDR1 is positively correlated with COL4A5 and COL4A6, but not other subunits (Figure 2a). These data suggest that basement membrane deposition and DDR1 overexpression were concomitant events in BQ-associated OSCC tissues.

In vitro, we asked whether overexpression of DDR1 could be initiated by continuous collagen stimulation. To test this, primary human oral keratinocyte (HOK) and immortalized keratinocyte cell lines (CGHNK6 and HaCaT) were treated with type I or type IV collagens, followed by RT-qPCR assay and western blot analysis. The results showed that both mRNA and protein level of DDR1 were increased by collagen treatment in all keratinocytes tested, suggesting that co-culture of keratinocytes with collagen increased DDR1 expression (Figure 2b and Figure S2).

### 2.3. DDR1 in OSCC Cells is Ligand-Independent and Constitutively Active

**Figure 3:**
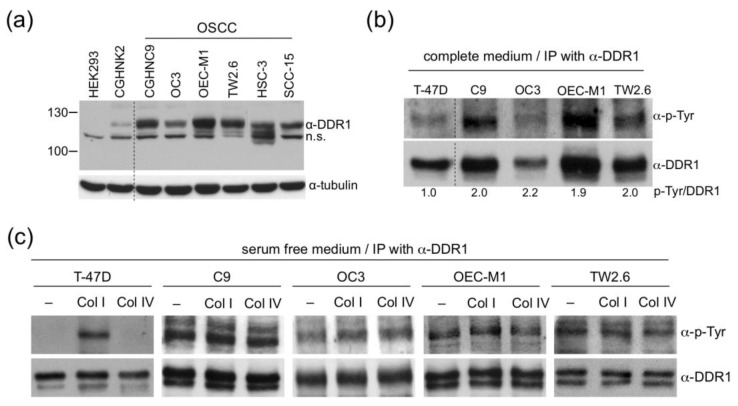
Constitutive phosphorylation of DDR1 in OSCC cells. (**a**) Western blot analysis of relative DDR1 expression levels in HEK293, CGHNK2, and six OSCC cells. Thirty μg protein lysates from each sample were used. α-tubulin served as an internal control. n.s., a nonspecific polypeptide cross-reactive to α-DDR1 antibody. (**b**) Total protein extracts of T-47D and OSCC cells were immunoprecipitated with the DDR1 antibody followed by western blot analysis using antibodies against p-Tyr or DDR1. The ratio of phosphorylated DDR1 intensity to that of total DDR1 was quantitated using ImageJ, with T-47D set to one. Similar results were obtained from two independent experiments; one set of data is shown. (**c**) T-47D and OSCC cells were cultured in a serum starved medium for 24 h followed by collagen stimulation (Col I or Col IV, 10 μg/mL) for an additional 24 h. The tyrosine-phosphorylation state of DDR1 was determined by immunoprecipitation-western blot analysis as described in (**b**).

To elucidate detailed molecular functions of DDR1 in BQ related OSCC, we collected four Taiwanese OSCC cell lines, namely CGHNC9 (abbreviated as C9 hereafter), OC3, OEC-M1, and TW2.6, from different regional institutions [36,37,38,39]. First, western blot analysis of DDR1 protein levels confirmed that OSCC cells expressed higher levels of DDR1 compared to that in the human embryonic kidney cell HEK293 and the oral keratinocyte CGHNK2 (Figure 3a and Figure S3). Next, to assess whether overexpressed DDR1 is autophosphorylated, the tyrosine phosphorylation states of DDR1 in C9, OC3, OEC-M1, and TW2.6 were monitored by immunoprecipitation-western blot analysis. The breast cancer cell line T-47D was included as a control because its DDR1 has been well characterized by Vogel et al. [40]. As depicted in Figure 3b, compared to T-47D, DDR1 in OSCC cells showed higher levels of tyrosine phosphorylation, suggesting that OSCC DDR1 is constitutively active in regular culture condition. Third, prior results showed that DDR1 phosphorylation was increased in T-47D cells treated with collagen I but not in cells treated with collagen IV [40]; thus, we performed a similar experiment for OSCC DDR1 to assess whether its tyrosine phosphorylation could be further augmented by either types of collagen. The results showed that, unlike T-47D, serum starvation was unable to completely abolish DDR1 phosphorylation in OSCC cells and neither collagen I nor IV could induce further DDR1 phosphorylation in OSCC cells (Figure 3c and Figure S4). Overall, the extent of DDR1 tyrosine phosphorylation correlates with DDR1 protein level, reinforcing the notion that DDR1 in these OSCC cells is constitutively phosphorylated. Combined, these results suggest that, in OSCC cells, DDR1 is constitutively active and unresponsive to further collagen stimulation. 

### 2.4. Constitutively Active DDR1 Contributes to Cell Growth and Clonogenicity

**Figure 4:**
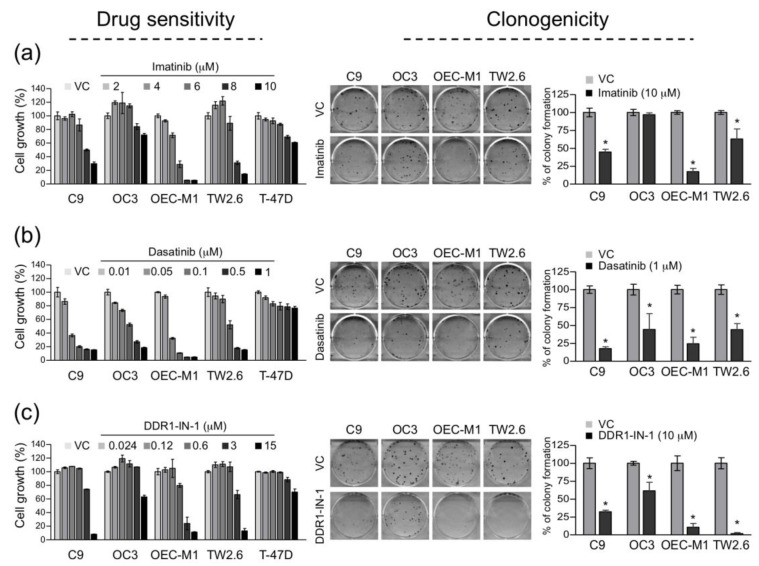
DDR1 kinase inhibitors suppressed OSCC cell growth and clonogenicity. Drug sensitivity (left part in each panel) was determined by treating cells with 2–10 μM imatinib. (**a**) 0.01–1 μM dasatinib, (**b**) or 0.024–15 μM DDR1-IN-1 (**c**) for 3 d, followed by WST-1 proliferation assay. VC, vehicle control (H_2_O for imatinib; DMSO for dasatinib and DDR1-IN-1). For each treatment, data are presented as mean ± SD from quadruplicate wells. Breast cancer cell line T-47D served as a control. Similar results were obtained from two independent experiments; one set of data is shown. Clonogenic survival assay (right part in each panel) was determined by treating cells in quadruplicate wells with 10 μM imatinib, (**a**) 1 μM dasatinib (**b**) or 10 μM DDR1-IN-1 (**c**) for 24 h, followed by cultivating cells in drug-free media for an additional 10–14 d. Representative photographs of crystal violet staining for each treatment are shown. Colony counts from two independent experiments are presented as mean ± SD, with the VC group set to 100%. * *p* < 0.05.

By targeting oncogenic tyrosine kinase activities, imatinib and dasatinib are two clinically approved small molecule inhibitors effective for patients with chronic myelogenous leukemia and acute lymphocytic leukemia. Noticeably, imatinib and dasatinib are also potent inhibitors for DDR1 [41,42,43]. On the other hand, DDR1-IN-1 is a recently developed inhibitor specific to DDR1 [44,45]. To inspect the efficacy of each drug against OSCC cell growth, serial dilutions of imatinib, dasatinib, and DDR1-IN-1 were added to OSCC culture media followed by a WST-1 assay. T-47D was included as a control since its activation of DDR1 requires collagen I stimulation (Figure 3c). As summarized in Figure 4, in general, all three drugs exhibited higher potency in restricting OSCC cell growth than the control cell line, which suggests that DDR1 might be a dominant oncogene in these OSCC cells. Additionally, clonogenic proliferation assay was used to test the efficacy of DDR1 inhibitors on OSCC cell survival. The results showed that imatinib efficiently inhibited colony formation of C9, OEC-M1, and TW2.6, whereas dasatinib and DDR1-IN-1 inhibited all of the four OSCC cells in clonogenic proliferation. Taken together, these results indicate that DDR1 kinase activity is crucial to OSCC cell growth and clonogenicity in vitro, a prosurvival or ‘oncogene addiction’ feature previously described in other cancer types (reviewed in [14]).

### 2.5. DDR1 is Involved in Collective Cell Invasion of OSCC TW2.6 Cells

**Figure 5:**
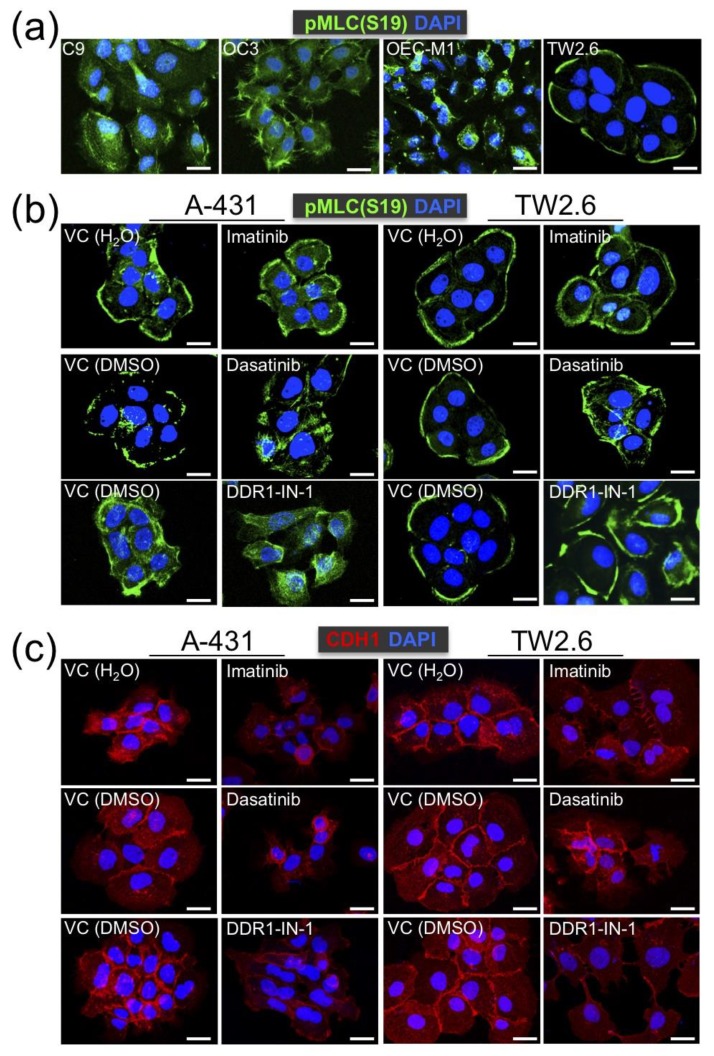
Kinase activity of DDR1 is involved in cohesive cell cluster formation. (**a**) Confocal-microscopic cell images of Ser19-phosphorylated myosin light chain (pMLC (S19)) staining (green) in the indicated OSCC cell lines. Nuclei were counterstained with DAPI (blue). Multi-cell cohesiveness, as evident by decreased pMLC (S19) staining at cell–cell contacts, was only observed in TW2.6 cells. (**b**) Confocal images of pMLC (S19) staining in A-431 (left) and TW2.6 (right) cells treated with vehicle control (VC) or DDR1 inhibitors (10 μM imatinib, 0.1 μM dasatinib, or 10 μM DDR1-IN-1). (**c**) Confocal images of CDH1 staining (red) in A-431 and TW2.6 treated with VC or DDR1 inhibitors. Scale bars, 20 μm.

Previously, Hidalgo-Carcedo et al. demonstrated that DDR1 participates in the collective migration of A-431 squamous cancer cells by coordinating the cell polarity regulators to reduce actomyosin activity at the cell–cell contact area [17]. To test whether OSCC DDR1 exerts similar functionality, immunostaining of an active myosin light chain, pMLC (Ser19), was performed to determine the actomyosin organization of OSCC cells on a 2D culture. We observed that cell cohesiveness was most evident in TW2.6, compared to that in C9, OC3 and OEC-M1 (Figure 5a). Next, A-431 and TW2.6 cells were studied in parallel with the same methodology. While multi-cell cohesiveness was prominent in both cells, cohesiveness was disrupted in either cell treated with DDR1 inhibitors, indicating that intact DDR1 kinase activity is crucial to multi-cell cohesion (Figure 5b). This result is consistent with the role of DDR1 in controlling appropriate actomyosin activity at the cell–cell contacts described before [17]. In addition, the reduction of CDH1 staining at the cellular junctions was concurrent with the blockade of DDR1 kinase activity (Figure 5c), reinforcing that DDR1 kinase activity is required for coherently holding multiple cells.

**Figure 6:**
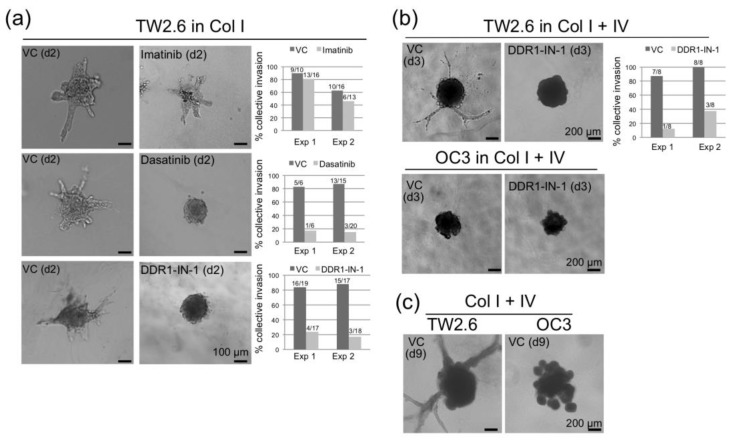
Kinase activity of DDR1 is involved in spheroid invasion. (**a**) Representative bright-field images of two-day TW2.6 cell spheroids embedded in collagen I gel matrix with vehicle control (VC) or DDR1 inhibitors (10 μM imatinib, 0.1 μM dasatinib, 10 μM DDR1-IN-1). In each assay, a protrusion with >100 μm extension from its spheroid central body was counted as a branch; a spheroid with at least two branches was scored positive for collective invasion. The results of two independent experiments are summarized on the right. The fraction on each graph bar denotes positive (numerator) and total (denominator) numbers of spheroids examined. (**b**) Bright-field images of three-day TW2.6 and OC3 spheroids embedded in collagen I and IV gel matrix with VC or 10 μM DDR1-IN-1. (**c**) Bright-field images of nine-day TW2.6 and OC3 spheroids embedded in collagen I and IV gel matrix with VC only.

An alternative approach to assess collective cell migration is spheroid invasion assay [27,46]. To this end, multicellular spheroid of TW2.6 was implanted into a 3D collagen gel matrix supplemented with a serum-free medium. Invasive cells emerged from the cell clusters, with astral outgrowing structures (branches) quantified as described by Andersen et al. [47]. OC3, a mesenchymal subtype of OSCC cell with low CDH1 and CDH2 expression, was included as a control. As expected, while no branching was observed in any OC3 spheroid, TW2.6 cells were able to invade collectively in either collagen type I alone (Figure 6a), or combination of collagen types I and IV (Figure 6b). Note that spheroid invasion was severely repressed by dasatinib and DDR1-IN-1, suggesting that the kinase activity of DDR1 is required during collective cell invasion. Interestingly, the branching process of TW2.6 continued to extend after a nine-day incubation, at which the OC3 spheroid was collapsed (Figure 6c).

### 2.6. DDR1 Specific Inhibitor, DDR1-IN-1, Suppressed ALI and PNI in TW2.6 Xenograft Tissues

**Figure 7:**
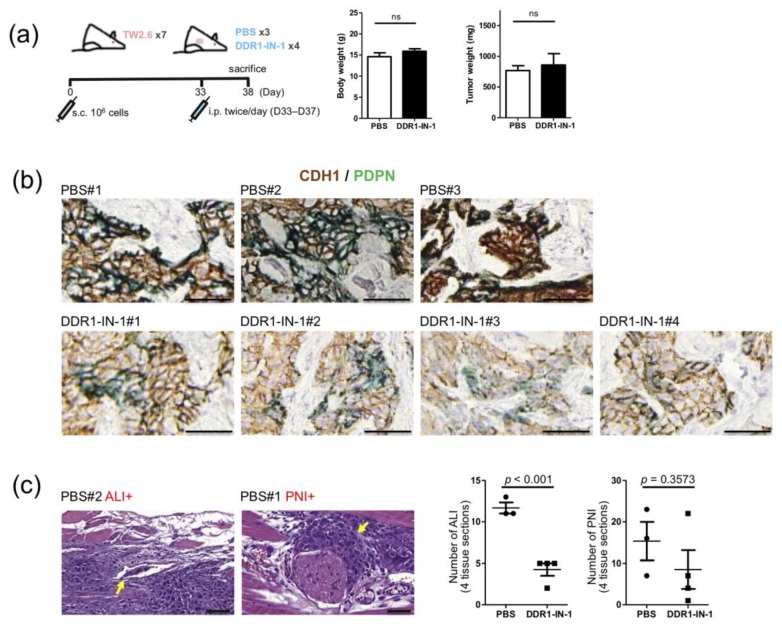
Suppression of collective TW2.6 cell invasion by DDR1-IN-1 in vivo. (**a**) (left) Schematic workflow of animal study. (right) Box plots show neither body weight nor tumor weight of mice in the control (PBS) and DDR1-IN-1 groups is statistically different. (**b**) Representative immunohistochemistry images of indicated TW2.6 xenograft tissues stained for CDH1 (brown) and PDPN (green). (**c**) Representative hematoxylin and eosin (H&E) stained xenograft sections with angiolymphatic invasion (ALI+) or perineural invasion (PNI+). Scale bars, 50 μm. Quantitative comparisons of ALI/PNI between PBS and DDR1-IN-1 mice are summarized on the right.

To explore the applicability of DDR1 inhibitor in blocking collective OSCC cell invasion in vivo, a TW2.6 derived xenograft mouse model was carried out by subcutaneous injection of TW2.6 cells to form tumor mass ~500 mm^3^ in size, followed by intraperitoneal injection of DDR1-IN-1 (25 mg/kg) for five consecutive days (Figure 7a). While neither body weight nor tumor weight showed statistical difference between the control and the DDR1-IN-1 mice, results of the CDH1/PDPN dual immunohistochemical stained sections from two groups were distinct. The intensities of CDH1 and PDPN were concomitantly decreased in xenograft tissues from mice administered with DDR1-IN-1, compared to that in tissues from the control mice (Figure 7b and Figure S5). This phenomenon is consistent with in vitro observations in that DDR1-IN-1 reduced CDH1 level (Figure 5c) and recapitulates the clinical finding that PDPN staining is strongly correlated with collective cell invasion (Figure 1c). Finally, quantitation of ALI and PNI for each xenograft revealed that DDR1-IN-1 was capable of reducing these two pathological features, of which ALI achieved statistical significance (*p* < 0.001) (Figure 7c). Taken together, these results suggest that DDR1-IN-1 could be an effective therapeutic adjuvant in disruption of collective OSCC cell invasion in vivo.

## 3. Discussion

Collagens are a group of ECM proteins crucial to the organization and shape of tissues. Under disease conditions, abnormal collagen deposition is attributable to initiate tissue fibrosis and cancer development [48]. The excessive deposition of collagen in the connective tissue is a key feature of oral premalignant and cancerous tissues in patients with BQ chewing habits [8,9,49]. In our NCKU-OrCA-40TN cohort, we noticed that five collagen subunits are upregulated genes in the tumor tissues compared to that in the normal counterparts, including COL1A1 (3.5×), COL4A1 (2.1×), COL4A2 (3.3×), COL4A5 (5.6×), and COL4A6 (9.2×). Among these, COL4A5 and COL4A6 were each statistically co-overexpressed with DDR1 in the 40 tumor tissues (Figure 2a). In vitro, cultivating primary or immortalized oral keratinocytes in the presence of collagen led to increased levels of DDR1 mRNA and protein (Figure 2b). Combined, it is tempting to hypothesize that collagen accumulation in injured tissue is a key driver for DDR1 expression in OSCC cells. Interestingly, Gross et al. demonstrated that loss of DDR1 expression in kidney glomerular epithelial cells delayed renal fibrosis in a mouse model of Alport syndrome, a hereditary collagen IV disease, suggesting that DDR1 and type IV collagen are critical drivers in renal fibrosis [50]. Whether the progression of oral cancer also involves similar pathologic interactions between DDR1 and type IV collagen is worth further investigation.

Overexpressed DDR1 was previously documented as a common survival driver for a panel of cancer cell lines derived from breast, pancreatic, and ovarian cancers [51]. Similarly, in OSCC cells, we showed that most of the overexpressed DDR1 are constitutively active and ligand-independent (Figure 3). Forced inhibition of DDR1 kinase activity in the OSCC cells greatly reduced cell growth and clonogenicity (Figure 4). These results imply that the OSCC cells might have been addicted to overexpressed DDR1 for their survival. In the same vein, targeting DDR1 alone or in combination with other chemotherapies have been suggested in several neoplasms, including metastatic colorectal cancer, K-RAS driven lung adenocarcinoma, and breast carcinoma [14,52,53,54].

Collective cell migration is a context-dependent process that is accompanied by three hallmarks: (1) cells remain connected during movement; (2) the traction and protrusion force for migration are generated by a multicellular organization of actin cytoskeleton; (3) the creation of a migration path in the tissue, through either secondary ECM installation (e.g. basement membrane) or clearing the track by stromal proteinases [27]. Growing evidence from analysis of patient samples indicates that collective cancer cell migration might be an efficient way for tumor cells to invade the underlying tissue and metastasize to other sites within the body [28,55].

Previously, collective migration of A-431 squamous cancer cells was linked to DDR1-directed low actomyosin activity at the cell–cell contact area [17]. By using 2D cell cohesiveness staining and 3D collagen gel matrix invasion assay, we showed that OSCC DDR1 exerts similar functionality in TW2.6 cells (Figure 5 and Figure 6). In addition, TW2.6 derived xenografts from mice treated with DDR1-IN-1 displayed significant reductions in ALI and PNI, two pathological features indicative of collective cell migration (Figure 7). Taken together, these results partially recapitulate the associations of DDR1 and PDPN with ALI detected in OSCC tumor tissues (Figure 1c). 

By using single cell RNA-seq analysis, Puram et al. recently established the first transcriptomic landscape of tumor ecosystems comprising ~6000 single cells from 23 head and neck squamous cell carcinoma specimens, including five matched lymph node metastases [56]. In their study, PDPN was consistently expressed in the leading edge or periphery region of malignant cell ‘nests’. These large intact tumor clusters were evident in both primary tumor and lymph node metastasis. Designated as partial epithelial-to-mesenchymal transition (p-EMT), this collective cell invasion-like phenotype is statistically associated with overexpression of PDPN, TGFBI, and SERPINE1, among others. Intriguingly, these three markers are also top-ranking genes associated with the ALI phenotype identified in our cohort (Table A1 and Figure A1), suggesting that ALI and p-EMT might share certain similarities. Furthermore, p-EMT was recapitulated as a dynamic and invasive process in vitro by using oral cancer cell line, SCC9. In vivo, the p-EMT program is likely regulated by stromal components residing in the microenvironment, including cancer associated fibroblasts and an array of ECM [56]. 

In summary, the present study provided two potential oncogenic roles of DDR1 in OSCC pathogenesis: prosurvival cell growth and collective cancer cell invasion. Tyrosine kinase activity of DDR1 is required for both malignant functions; thus, clinically approved DDR1 kinase inhibitors could be considered as novel or adjuvant therapies for oral cancer treatments.

## 4. Materials and Methods

### 4.1. Cell Culture and Drug Sensitivity Assay

CGHNK2, CGHNK6, CGHNC9 [39], OC3 [38], OEC-M1 [36], TW2.6 [37] were kindly provided by researchers at different Institutions in Taiwan. A-431 (CRL-1555, ATCC) and T-47D (HTB-133, ATCC) was kindly provided by Dr. Dan Robinson (MCTP, University of Michigan, Ann Arbor, MI, USA). The primary human oral keratinocytes were purchased from ScienCell Research Laboratories (Carlsbad, CA, USA) and Rheinwald Lab [57]. All cells were cultivated in specified media as described in the original literature or manufacturer’s instructions. The cells were grown in a humidified 37 °C incubator with 5% CO_2_. The drug sensitivity of imatinib, dasatinib, and DDR1-IN-1 was determined by using a WST-1 assay (Roche, Indianapolis, IN, USA). Briefly, 2,000 cells were seeded in a 96-well plate overnight before drug treatments. The cells were incubated with indicated concentrations of drugs for three days. Ten μL of WST-1 reagent was added to one well of culture containing 100 μL of medium. Two hours after incubation at 37 °C, the microplate was gently mixed for 1 min and the absorbance was measured by using an ELISA reader (Infinite M200, Tecan, Männedorf, Switzerland) at 450 nm with a reference wavelength of 690 nm.

### 4.2. Antibodies and Reagents

The antibodies and reagents used in this study were purchased from the following sources: anti-CDH1 (#3195) and anti-p-MLC (S19) (#3671) was from Cell Signaling (Danvers, MA, USA); anti-α-tubulin (#05-829) and anti-p-Tyr (4G10) (#16-316) was from Millipore (Billerica, MA, USA); anti-DDR1 was from Santa Cruz Biotechnology (sc-532, Santa Cruz, CA, USA); anti-PDPN was from Biocare Medical (CM 266, Pacheco, CA, USA); imatinib mesylate was from Sigma-Aldrich (SML1027, St. Louis, MO, USA); dasatinib was from Selleckchem (S1021, Houston, TX, USA). DDR1-IN-1 was from MedChemExpress (HY-13979, Monmouth Junction, NJ, USA). Type I and type IV collagens were from BD Biosciences (San Jose, CA, USA). 

### 4.3. Reverse Transcription-Quantitative Real-Time PCR (RT-qPCR)

One microgram of RNA was reverse transcribed to cDNAs by using SuperScript III reverse transcriptase (Invitrogen, Carlsbad, CA) in a 20 μL reaction mixture. For each real-time PCR reaction, 4 μL diluted cDNAs templates were thoroughly mixed with 1 μL primer mix (2 μM) and 5 μL Power SYBR Green MasterMix (Applied Biosystems, Foster City, CA, USA). The reaction was conducted and detected by StepOne Plus Real-Time PCR system according to manufacturer’s instructions (Applied Biosystems, Foster City, CA, USA). For each sample, the expression level of DDR1 was normalized to that of glyceraldehyde-3-phosphate dehydrogenase (GAPDH) using the ΔCT method. The DDR1 primer sequences: forward 5′-GGCCAAACCCACCAACAC-3′; reverse 5′-AACAATGTCAGCCTCGGCATA-3′.

### 4.4. Western Blot Analysis and Immunoprecipitation

The cells were lysed in an RIPA buffer (50 mM Tris-HCl, pH 8, 150 mM NaCl, 0.1% Nonidet P-40, 0.5% sodium deoxycholate, 0.1% SDS, 1 mM phenylmethylsulfonyl fluoride, 200 μM sodium orthovanadate, 1× protease inhibitors, 1× phosphatase inhibitors). The protein concentration was measured spectrophotometrically at 562 nm by using the BCA Pierce^TM^ protein assay kit (Thermo Fisher Scientific, Waltham, MA, USA). For an immunoprecipitation assay, the cells were lysed in cold EBC lysis buffer (50 mM Tris-HCl, pH 7.4, 0.5% NP-40, 120 mM NaCl, 50 mM NaF, 1 mM phenylmethylsulfonyl fluoride, 200 μM sodium orthovanadate, 1× protease inhibitors, 1× phosphatase inhibitors). The protein extracts were gently mixed with anti-DDR1 bound Dynabeads-Protein G (Invitrogen, Carlsbad, CA, USA) on a nutator with continuous rotation at 4 °C overnight. After magnetic separation, the Dynabeads were washed thoroughly with washing buffer three times followed by immunocomplex elution. The cell lysates or the eluted immunocomplex was subjected to standard western blot analysis as described previously [58].

### 4.5. Clonogenic Survival Assay

Cells were cultured in six-well plates (250 cells per well for C9; 100 cells per well for OC3, OEC-M1, TW2.6) overnight followed by 10 μM imatinib, 1 μM dasatinib or 10 μM DDR1-IN-1 treatment for 24 h. After washed with PBS twice, cells were maintained in drug-free medium for an additional 10 to 14 d. Cells were fixed with 70% ethanol for 30 min at 4 °C, stained with 1% crystal violet for 30 min, and rinsed with water thoroughly. The stained colonies (>50 cells) were counted under a microscope. The colony count for each treatment was presented as mean ± SD derived from quadruple wells.

### 4.6. Immunofluorescence Assay

The cells grown on glass coverslips were fixed with 1% paraformaldehyde for 10 min and permeabilized with 0.1% Triton X-100 in PBS for 5 min. The samples were blocked with 1% BSA in PBS for 30 min before incubation with anti-p-MLC (S19) or anti-CDH1 in blocking solution overnight at 4 °C. After washing with PBS, the samples were incubated with secondary antibodies (#111-095-144 (FITC), #111-295-045 (Rhodamine), Jackson ImmunoResearch Laboratories, West Grove, PA, USA) for 1 h at room temperature. After DAPI staining for the nuclei, the slides were mounted with Prolong Gold (P36930; Invitrogen, Carlsbad, CA, USA). The images were acquired with a Leica TCS SP5 II confocal microscope and processed with LAS AF software (Leica, Wetzlar, Germany).

### 4.7. Spheroid Invasion Assays

Distinguished by collagen types, two protocols were used. In Figure 6a, only collagen I was used as a matrix. Briefly, 1500 TW2.6 cells were seeded in a 1% agar-coated 96-well round bottom plate to form aggregated spheroids. After 48 h, spheroids resuspended in serum free medium were mixed with an equal volume of bovine type I collagen (2.4 mg/mL, pH 7.5). The spheroid-collagen mixture (in 400 μL) was seeded into a 24-well plate, incubated for 30 min at 37 °C, followed by overlaying 100 μL culture medium with or without DDR1 inhibitors on top of the solidified mixture for collective cell invasion (48 h). In Figure 6b,c, Cultrex 96 Well 3D Spheroid BME Cell Invasion Assay kit (#3500-096-K; Trevigen, Gaithersburg, MD, USA) was used, in which collagen I and -IV were both included as the invasion matrix. In this assay, 1500 OC3 or TW2.6 cells resuspended in spheroid formation solution were seeded in a 96-well round bottom plate to form aggregated spheroids (72 h), followed by adding invasion matrix with or without DDR1-IN-1 for collective cell invasion (72 h). For both methods, bright-field spheroids images were captured by using an Olympus IX73 microscope. A spheroid with at least two branches extending from its central body (> 100 μm) was scored positive as described by Andersen et al. [47]. 

### 4.8. Dual Immunohistochemical Staining

OSCC sections were dewaxed, rehydrated and incubated with Trilogy^TM^ (Cell Marque, CA, USA) at 121 °C for 10 min to unmask antigens. At room temperature (RT), the slides were immersed in 3% hydrogen peroxide for 15 min to quench endogenous peroxidase activity; 1% bovine serum albumin was used for 60 min to block nonspecific antigenic sites. Slides were incubated with anti-PDPN and anti-DDR1 (Figure 1c) or anti-PDPN and anti-CDH1 at 4 °C overnight (Figure 7b). After washing with TBS, slides were incubated with alkaline phosphatase (AP)- and horseradish peroxidase (HRP)-conjugated secondary antibodies (MRCT523, Biocare Medical) for 30 min at RT. The color development of tissue sections was conducted by using chromagen StayGreen for AP (Abcam, Cambridge, UK) and diaminobenzidine (Agilent, CA, USA) for HRP. The slides were scanned by a Pannoramic MIDI scanner (3DHISTECH, Budapest, Hungary). This study was reviewed and approved by the Research Ethics Committee of National Health Research Institutes (Protocol No: EC1040407-E) for the use of clinical samples. 

### 4.9. Animal Experiment

For each mouse, one million mycoplasma-free TW2.6 cells mixed with equal volume of Corning^®^ Matrigel^®^ matrix (Corning, NY, USA) was subcutaneously implanted into the flank of an 11 week-old NOG (National Institute of Infectious Diseases and Vaccinology, Taiwan). When the tumor mass reached 500 mm^3^, mice were divided into PBS (*n* = 3) and DDR1-IN-1 (*n* = 4) groups, for intraperitoneal injection of PBS and DDR1-IN-1 (25 mg/kg), respectively. PBS or DDR1-IN-1 were administered twice a day, five days consecutively, followed by euthanization of mice and tumor collection. All procedures were approved by the Institutional Animal Care and Use Committee of National Health Research Institutes, Taiwan (Protocol No: NHRI-IACUC-106057-M1-A).

### 4.10. Bioinformatics and Statistical Analysis

The normalized microarray dataset GSE37991, comprising an expression matrix of 17,544 genes × 80 samples (40T + 40N) [31], were downloaded from the NCBI GEO portal and analyzed by various R (3.6.1) packages in a Bioconductor (3.10). Specifically, *Limma* was used to identify differentially expressed genes (DEGs) accountable for angiolymphatic invasion (ALI) [33] (Table A1). The results were highly similar to DEGs identified by *GSEA* [32] (Figure 1a). Selected DEGs (FC > 1.5) were subjected to gene ontology and pathway enrichment analyses by *DAVID* (v6.8) [34] (Figure 1b). The Pearson correlation test of DDR1 and collagen subunits were evaluated by *cor.test* (Figure 2a). For the rest of the study, the statistical analysis was carried out by Prism 5.0 (GraphPad Software). Specifically, an unpaired two-tailed t-test was applied to evaluate the results in RT-qPCR (Figure 2b), clonogenicity assay (Figure 4), and quantitation of ALI and PNI (Figure 7c). Differences were considered significant when *p* < 0.05. 

## 5. Conclusions

Excessive deposition of collagen in the connective tissue is an adverse feature of oral cancer and premalignant disorders. Through meta-analysis of a microarray dataset comprising 40 OSCC tissues, we showed that co-overexpression of DDR1 and COL4A5 or COL4A6 is statistically significant. In vitro elevated DDR1 mRNA and protein levels were observed by treating oral keratinocytes with collagen of fibrillar or basement membrane types. Overexpressed OSCC DDR1 is ligand-independent and constitutively phosphorylated. OSCC cells treated with DDR1 kinase inhibitors displayed reduced cell growth and clonogenicity. DDR1 kinase activity in the OSCC TW2.6 cell is required for multicellular cohesiveness, spheroid invasion, and collective cell invasion in xenograft tissues. Taken together, these data strongly suggest that constitutively active DDR1 is crucial to collective OSCC cell invasion in vivo, including angiolymphatic invasion. Since DDR1 is a druggable target, our results provide an alternative therapeutic approach in blocking tumor cell growth and metastasis for oral cancer patients. 

## Supplementary Materials

The following are available online at https://www.mdpi.com/2072-6694/12/4/841/s1, Figure S1: Two magnifications (400× and 800×) of IHC images in Figure 1c, Figure S2: Uncropped scans of Western blots in Figure 2b, Figure S3: Uncropped scans of Western blots in Figure 3a,b, Figure S4: Uncropped scans of Western blots in Figure 3c, Figure S5: Two magnifications (400× and 800×) of IHC images in Figure 7b.

## Appendix A

**Table A1:** Top 90 genes differentially expressed between ALI-Yes and ALI-No identified by *Limma topTable* function.

Gene SYMBOL	Fold ^1^ (Yes/No)	Probability ^2^	Gene Symbol	Fold (Yes/No)	Probability	Gene Symbol	Fold (Yes/No)	Probability
LDHD	0.351	65.7	MMP3	1.346	19.0	GRHL3	0.481	11.9
SORCS2	2.233	62.9	TRIM56	1.241	19.0	LRRC73	1.438	11.8
NELL2	0.390	60.7	AAMDC	1.420	18.9	FBXO2	1.878	11.7
ACSF2	1.778	52.5	TMPRSS7	2.135	18.9	CYB561A3	1.388	11.2
MMP1	1.457	45.7	ITGA3	1.701	18.6	ITGA2	1.221	11.1
SERPINE1	2.103	45.6	TNK1	1.717	18.2	IQCE	1.415	11.1
DDR1	1.569	45.4	CHST3	1.232	17.8	ZNF114	1.927	11.0
CHST15	1.304	44.3	MCOLN3	2.266	17.0	PRODH	0.414	11.0
GRINA	1.257	38.5	HECTD1	0.877	16.5	ME1	0.758	10.8
CCDC30	1.313	38.3	PTGER1	0.680	15.8	NBPF3	1.539	10.8
ZNF704	2.084	33.4	PPP2R2B	2.034	15.6	ASB4	0.806	10.6
RNF5P1	1.425	33.3	GPR107	1.662	15.5	LYRM1	0.910	10.6
APCDD1L	2.075	32.3	SLC16A9	0.595	15.4	CYP4F22	0.276	10.6
CYP24A1	1.540	31.3	MCRIP1	1.522	15.1	XYLT2	1.176	10.4
REPS2	1.951	29.1	DLL1	1.428	14.8	KLC4	1.482	10.2
CAMK2N1	1.613	27.1	CDCA7L	1.440	14.8	CGB5	2.487	10.2
TINAGL1	1.751	27.1	FOXC2	1.850	14.6	EVA1A	1.926	10.1
KLK14	0.269	26.1	RTN4RL2	1.204	14.4	MDFIC	0.780	10.0
TGFBI	1.192	23.2	SPECC1	1.479	14.0	PRKCH	0.813	9.9
NOS3	0.606	21.3	NCS1	1.581	13.6	SLC2A6	1.359	9.8
ZDHHC21	0.625	21.1	CCM2	1.197	13.5	EFHB	0.487	9.8
FSTL3	1.753	20.8	UPK1A	0.349	13.3	EPHB2	2.235	9.8
AUH	1.562	20.7	GLB1	1.318	13.1	FREM1	0.681	9.6
ITGB4	1.320	20.3	SYK	0.633	13.0	MFSD6	0.789	9.5
C4orf26	2.344	20.2	GLTP	0.795	12.8	GUCA2B	0.728	9.5
GPAT3	0.680	20.1	COL4A6	1.842	12.7	ZBTB18	0.889	9.4
RRAS	1.308	19.6	BMP8B	1.412	12.5	NELFB	1.364	9.2
GPRC5D	0.672	19.4	PDPN	1.585	12.5	LYN	1.207	9.1
SEMA3G	0.731	19.3	HPSE	0.636	12.1	ABHD12	0.812	9.0
PRRC2A	1.329	19.2	SLC43A2	1.763	12.0	TMEM63C	0.565	8.9

^1^ Transformed from log2-fold-change (LogFC) ^2^ Calculated from *B* statistic ((EXP(*B*)/(1 + EXP(*B*))) ∗ 100).

## References

[1] Leemans C.R., Braakhuis B.J., Brakenhoff R.H. (2011). The molecular biology of head and neck cancer. Nat. Rev. Cancer.

[2] Taiwan MOHW Cancer Registry Annual Cancer Report, Ministry of Health and Welfare, Taiwan. http://tcr.cph.ntu.edu.tw.

[3] Umar A., Dunn B.K., Greenwald P. (2012). Future directions in cancer prevention. Nat. Rev. Cancer.

[4] International Agency for Research on Cancer (2004). Betel-Quid and Areca-Nut Chewing and Some Areca-Nut-Derived Nitrosamines.

[5] Sharan R.N., Mehrotra R., Choudhury Y., Asotra K. (2012). Association of betel nut with carcinogenesis: Revisit with a clinical perspective. PLoS ONE.

[6] Chang M.C., Chiang C.P., Lin C.L., Lee J.J., Hahn L.J., Jeng J.H. (2005). Cell-mediated immunity and head and neck cancer: With special emphasis on betel quid chewing habit. Oral Oncol..

[7] Tsai W.C., Tsai S.T., Ko J.Y., Jin Y.T., Li C., Huang W., Young K.C., Lai M.D., Liu H.S., Wu L.W. (2004). The mRNA profile of genes in betel quid chewing oral cancer patients. Oral Oncol..

[8] Shieh D.H., Chiang L.C., Shieh T.Y. (2003). Augmented mRNA expression of tissue inhibitor of metalloproteinase-1 in buccal mucosal fibroblasts by arecoline and safrole as a possible pathogenesis for oral submucous fibrosis. Oral Oncol..

[9] Prime S.S., Davies M., Pring M., Paterson I.C. (2004). The role of TGF-beta in epithelial malignancy and its relevance to the pathogenesis of oral cancer (part II). Crit. Rev. Oral. Biol. Med..

[10] Rajalalitha P., Vali S. (2005). Molecular pathogenesis of oral submucous fibrosis-a collagen metabolic disorder. J. Oral Pathol. Med..

[11] Khan I., Kumar N., Pant I., Narra S., Kondaiah P. (2012). Activation of TGF-beta pathway by areca nut constituents: A possible cause of oral submucous fibrosis. PLoS ONE.

[12] Eswaramoorthy R., Wang C.K., Chen W.C., Tang M.J., Ho M.L., Hwang C.C., Wang H.M., Wang C.Z. (2010). DDR1 regulates the stabilization of cell surface E-cadherin and E-cadherin-mediated cell aggregation. J. Cell Physiol..

[13] Huang Y., Arora P., McCulloch C.A., Vogel W.F. (2009). The collagen receptor DDR1 regulates cell spreading and motility by associating with myosin IIA. J. Cell Sci..

[14] Valiathan R.R., Marco M., Leitinger B., Kleer C.G., Fridman R. (2012). Discoidin domain receptor tyrosine kinases: New players in cancer progression. Cancer Metastasis Rev..

[15] Lemeer S., Bluwstein A., Wu Z., Leberfinger J., Muller K., Kramer K., Kuster B. (2012). Phosphotyrosine mediated protein interactions of the discoidin domain receptor 1. J. Proteom..

[16] Vogel W.F., Abdulhussein R., Ford C.E. (2006). Sensing extracellular matrix: An update on discoidin domain receptor function. Cell. Signal..

[17] Hidalgo-Carcedo C., Hooper S., Chaudhry S.I., Williamson P., Harrington K., Leitinger B., Sahai E. (2011). Collective cell migration requires suppression of actomyosin at cell-cell contacts mediated by DDR1 and the cell polarity regulators Par3 and Par6. Nat. Cell Biol..

[18] Shimada K., Nakamura M., Ishida E., Higuchi T., Yamamoto H., Tsujikawa K., Konishi N. (2008). Prostate cancer antigen-1 contributes to cell survival and invasion though discoidin receptor 1 in human prostate cancer. Cancer Sci..

[19] Yamamoto E., Kohama G., Sunakawa H., Iwai M., Hiratsuka H. (1983). Mode of invasion, bleomycin sensitivity, and clinical course in squamous cell carcinoma of the oral cavity. Cancer.

[20] Cheung K.J., Gabrielson E., Werb Z., Ewald A.J. (2013). Collective invasion in breast cancer requires a conserved Basal epithelial program. Cell.

[21] Peglion F., Llense F., Etienne-Manneville S. (2014). Adherens junction treadmilling during collective migration. Nat. Cell Biol..

[22] Hirata E., Park D., Sahai E. (2014). Retrograde flow of cadherins in collective cell migration. Nat. Cell Biol..

[23] Li C.F., Chen J.Y., Ho Y.H., Hsu W.H., Wu L.C., Lan H.Y., Hsu D.S., Tai S.K., Chang Y.C., Yang M.H. (2019). Snail-induced claudin-11 prompts collective migration for tumour progression. Nat. Cell Biol..

[24] Retzbach E.P., Sheehan S.A., Nevel E.M., Batra A., Phi T., Nguyen A.T.P., Kato Y., Baredes S., Fatahzadeh M., Shienbaum A.J. (2018). Podoplanin emerges as a functionally relevant oral cancer biomarker and therapeutic target. Oral Oncol..

[25] Wicki A., Lehembre F., Wick N., Hantusch B., Kerjaschki D., Christofori G. (2006). Tumor invasion in the absence of epithelial-mesenchymal transition: Podoplanin-mediated remodeling of the actin cytoskeleton. Cancer Cell.

[26] Dos Santos Almeida A., Oliveira D.T., Pereira M.C., Faustino S.E., Nonogaki S., Carvalho A.L., Kowalski L.P. (2013). Podoplanin and VEGF-C immunoexpression in oral squamous cell carcinomas: Prognostic significance. Anticancer Res..

[27] Friedl P., Gilmour D. (2009). Collective cell migration in morphogenesis, regeneration and cancer. Nat. Rev. Mol. Cell Biol..

[28] Slattum G.M., Rosenblatt J. (2014). Tumour cell invasion: An emerging role for basal epithelial cell extrusion. Nat. Rev. Cancer.

[29] Tsai W.-H., Chen Y.-L., Chen H.-C., Cheng A.J., Chang K.-Y., Chu P.-Y., Hsiao J.-R., Chang J.-Y., Lin S.-F. (2012). Overexpression of Discoidin Domain Receptor 1 (DDR1) in oral squamous cell carcinoma. Eur. J. Cancer.

[30] Chou S.T., Peng H.Y., Mo K.C., Hsu Y.M., Wu G.H., Hsiao J.R., Lin S.F., Wang H.D., Shiah S.G. (2019). MicroRNA-486-3p functions as a tumor suppressor in oral cancer by targeting DDR1. J. Exp. Clin. Cancer Res..

[31] Lee C.H., Wong T.S., Chan J.Y., Lu S.C., Lin P., Cheng A.J., Chen Y.J., Chang J.S., Hsiao S.H., Leu Y.W. (2013). Epigenetic regulation of the X-linked tumour suppressors BEX1 and LDOC1 in oral squamous cell carcinoma. J. Pathol..

[32] Subramanian A., Tamayo P., Mootha V.K., Mukherjee S., Ebert B.L., Gillette M.A., Paulovich A., Pomeroy S.L., Golub T.R., Lander E.S. (2005). Gene set enrichment analysis: A knowledge-based approach for interpreting genome-wide expression profiles. Proc. Natl. Acad. Sci. USA.

[33] Ritchie M.E., Phipson B., Wu D., Hu Y., Law C.W., Shi W., Smyth G.K. (2015). limma powers differential expression analyses for RNA-sequencing and microarray studies. Nucleic Acids Res..

[34] Huang da W., Sherman B.T., Lempicki R.A. (2009). Systematic and integrative analysis of large gene lists using DAVID bioinformatics resources. Nat. Protoc..

[35] Kawaguchi H., El-Naggar A.K., Papadimitrakopoulou V., Ren H., Fan Y.H., Feng L., Lee J.J., Kim E., Hong W.K., Lippman S.M. (2008). Podoplanin: A novel marker for oral cancer risk in patients with oral premalignancy. J. Clin. Oncol..

[36] Chen J.H., Lim J.S., Shyu K.W., Meng C.L. (1988). Direct cytotoxicity of garlic on human oral cancer cells. Zhonghua Ya Yi Xue Hui Za Zhi.

[37] Kok S.H., Hong C.Y., Lin S.K., Lee J.J., Chiang C.P., Kuo M.Y. (2007). Establishment and characterization of a tumorigenic cell line from areca quid and tobacco smoke-associated buccal carcinoma. Oral Oncol..

[38] Lin S.C., Liu C.J., Chiu C.P., Chang S.M., Lu S.Y., Chen Y.J. (2004). Establishment of OC3 oral carcinoma cell line and identification of NF-kappa B activation responses to areca nut extract. J. Oral Pathol. Med..

[39] Lu Y.C., Chen Y.J., Wang H.M., Tsai C.Y., Chen W.H., Huang Y.C., Fan K.H., Tsai C.N., Huang S.F., Kang C.J. (2012). Oncogenic function and early detection potential of miRNA-10b in oral cancer as identified by microRNA profiling. Cancer Prev. Res..

[40] Vogel W., Gish G.D., Alves F., Pawson T. (1997). The discoidin domain receptor tyrosine kinases are activated by collagen. Mol. Cell.

[41] Bantscheff M., Eberhard D., Abraham Y., Bastuck S., Boesche M., Hobson S., Mathieson T., Perrin J., Raida M., Rau C. (2007). Quantitative chemical proteomics reveals mechanisms of action of clinical ABL kinase inhibitors. Nat. Biotechnol..

[42] Day E., Waters B., Spiegel K., Alnadaf T., Manley P.W., Buchdunger E., Walker C., Jarai G. (2008). Inhibition of collagen-induced discoidin domain receptor 1 and 2 activation by imatinib, nilotinib and dasatinib. Eur. J. Pharmacol..

[43] Rix U., Hantschel O., Durnberger G., Remsing Rix L.L., Planyavsky M., Fernbach N.V., Kaupe I., Bennett K.L., Valent P., Colinge J. (2007). Chemical proteomic profiles of the BCR-ABL inhibitors imatinib, nilotinib, and dasatinib reveal novel kinase and nonkinase targets. Blood.

[44] Canning P., Tan L., Chu K., Lee S.W., Gray N.S., Bullock A.N. (2014). Structural mechanisms determining inhibition of the collagen receptor DDR1 by selective and multi-targeted type II kinase inhibitors. J. Mol. Biol..

[45] Kim H.G., Tan L., Weisberg E.L., Liu F., Canning P., Choi H.G., Ezell S.A., Wu H., Zhao Z., Wang J. (2013). Discovery of a potent and selective DDR1 receptor tyrosine kinase inhibitor. ACS Chem. Biol..

[46] Justus C.R., Leffler N., Ruiz-Echevarria M., Yang L.V. (2014). In vitro cell migration and invasion assays. J. Vis. Exp..

[47] Andersen K., Mori H., Fata J., Bascom J., Oyjord T., Maelandsmo G.M., Bissell M. (2011). The metastasis-promoting protein S100A4 regulates mammary branching morphogenesis. Dev. Biol..

[48] Lu P., Takai K., Weaver V.M., Werb Z. (2011). Extracellular matrix degradation and remodeling in development and disease. Cold Spring Harbor Perspect. Biol..

[49] Lu S.L., Reh D., Li A.G., Woods J., Corless C.L., Kulesz-Martin M., Wang X.J. (2004). Overexpression of transforming growth factor beta1 in head and neck epithelia results in inflammation, angiogenesis, and epithelial hyperproliferation. Cancer Res..

[50] Gross O., Girgert R., Beirowski B., Kretzler M., Kang H.G., Kruegel J., Miosge N., Busse A.C., Segerer S., Vogel W.F. (2010). Loss of collagen-receptor DDR1 delays renal fibrosis in hereditary type IV collagen disease. Matrix Biol..

[51] Marcotte R., Brown K.R., Suarez F., Sayad A., Karamboulas K., Krzyzanowski P.M., Sircoulomb F., Medrano M., Fedyshyn Y., Koh J.L. (2012). Essential gene profiles in breast, pancreatic, and ovarian cancer cells. Cancer Discov..

[52] Ambrogio C., Gomez-Lopez G., Falcone M., Vidal A., Nadal E., Crosetto N., Blasco R.B., Fernandez-Marcos P.J., Sanchez-Cespedes M., Ren X. (2016). Combined inhibition of DDR1 and Notch signaling is a therapeutic strategy for KRAS-driven lung adenocarcinoma. Nat. Med..

[53] Jeitany M., Leroy C., Tosti P., Lafitte M., Le Guet J., Simon V., Bonenfant D., Robert B., Grillet F., Mollevi C. (2018). Inhibition of DDR1-BCR signalling by nilotinib as a new therapeutic strategy for metastatic colorectal cancer. EMBO Mol. Med..

[54] Jing H., Song J., Zheng J. (2018). Discoidin domain receptor 1: New star in cancer-targeted therapy and its complex role in breast carcinoma. Oncol. Lett..

[55] Cheung K.J., Ewald A.J. (2016). A collective route to metastasis: Seeding by tumor cell clusters. Science.

[56] Puram S.V., Tirosh I., Parikh A.S., Patel A.P., Yizhak K., Gillespie S., Rodman C., Luo C.L., Mroz E.A., Emerick K.S. (2017). Single-Cell Transcriptomic Analysis of Primary and Metastatic Tumor Ecosystems in Head and Neck Cancer. Cell.

[57] Lindberg K., Rheinwald J.G. (1990). Three distinct keratinocyte subtypes identified in human oral epithelium by their patterns of keratin expression in culture and in xenografts. Differentiation.

[58] Tsai W.H., Wang P.W., Lin S.Y., Wu I.L., Ko Y.C., Chen Y.L., Li M., Lin S.F. (2012). Ser-634 and Ser-636 of Kaposi’s Sarcoma-Associated Herpesvirus RTA are Involved in Transactivation and are Potential Cdk9 Phosphorylation Sites. Front. Microbiol..

[59] Goldman M., Craft B., Hastie M., Repečka K., McDade F., Kamath A., Banerjee A., Luo Y., Rogers D., Brooks A.N. (2019). The UCSC Xena platform for public and private cancer genomics data visualization and interpretation. bioRxiv.

