# Supplementary material for: Discoidin Domain Receptor-1 (DDR1) is Involved in Angiolymphatic Invasion in Oral Cancer
Source: Cancers (Basel). 2020 Mar 31;12(4):841. doi: 10.3390/cancers12040841 (PMC7226486; doi:10.3390/cancers12040841)
Supplement: Supplementary file 1 [file cancers-12-00841-s001.pdf]

Supplementary Materials: Discoidin Domain Receptor-1 (DDR1) is Involved in Angiolymphatic Invasion in Oral Cancer

Yu-Lian Chen, Wan-Hua Tsai, Ying-Chieh Ko, Ting-Yu Lai, Ann-Joy Cheng, Shine-Gwo Shiah, Jenn-Ren Hsiao, Jang-Yang Chang and Su-Fang Lin

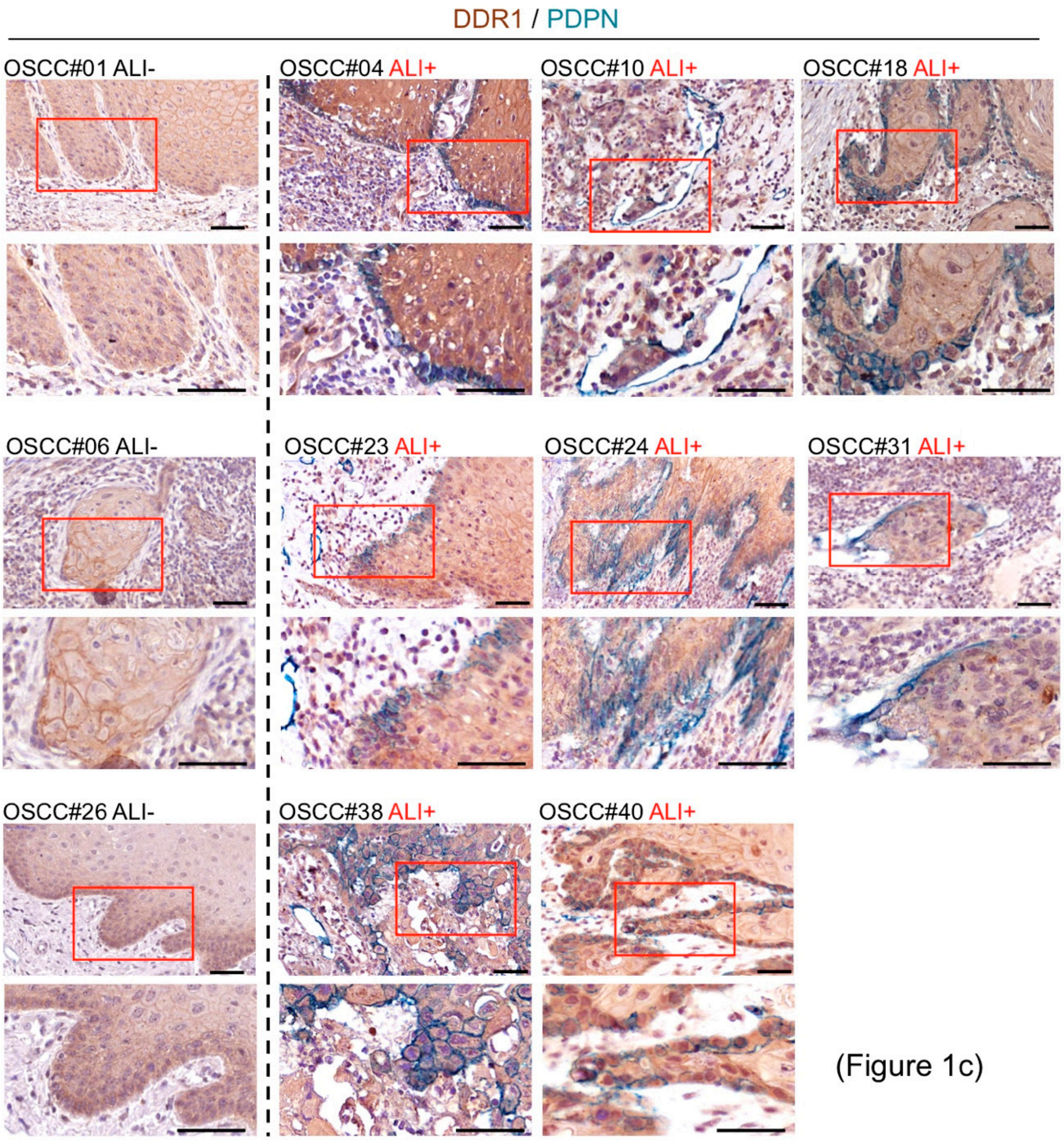

Figure S1. Two magnifications (400× and 800×) of immunohistochemistry images in Figure 1c. Scale bars, 50 μm.

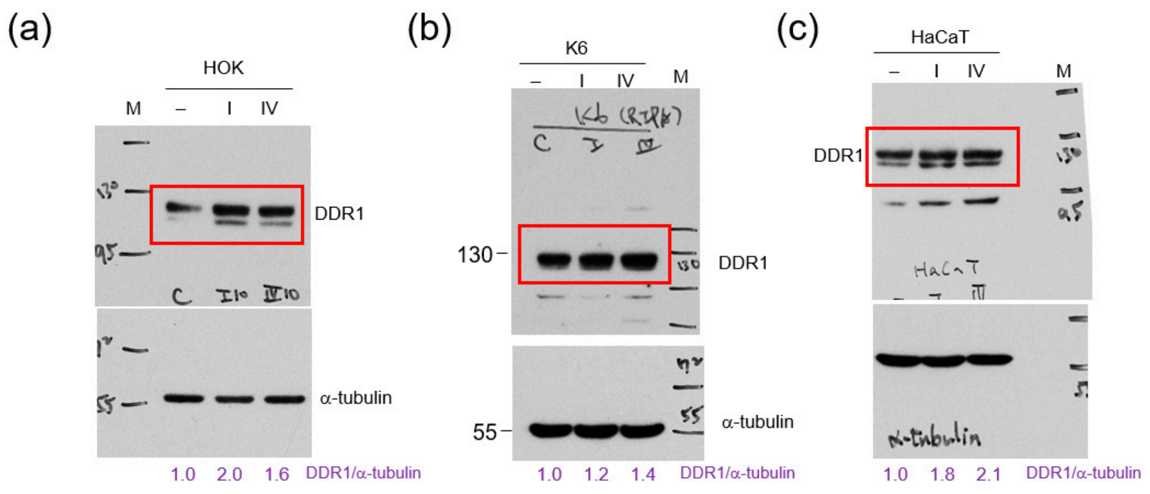

Figure S2. Uncropped scans of Western blots in Figure 2b. In each lane, ratio of DDR1 intensity to that of α-tubulin was quantitated by using ImageJ. The ratios in each untreated sample were set to 1.

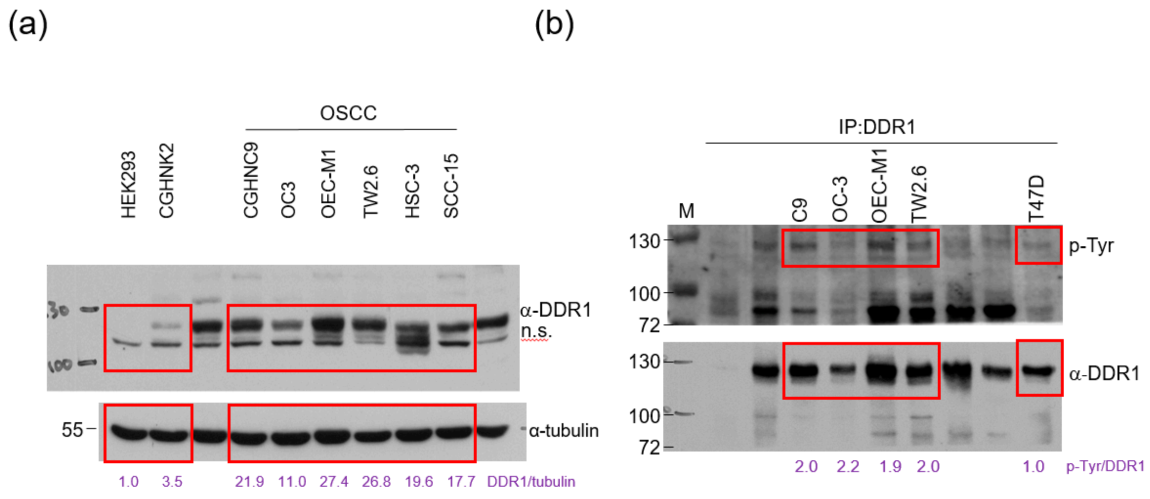

Figure S3. Uncropped scans of Western blots in Figure 3a and 3b. For each sample, ratio of DDR1 intensity to that of α-tubulin (a); phosphorylated DDR1 intensity to that of total DDR1 (b), was quantitated by using ImageJ. The ratios in HEK293 (a) and T47D (b) were set to 1.

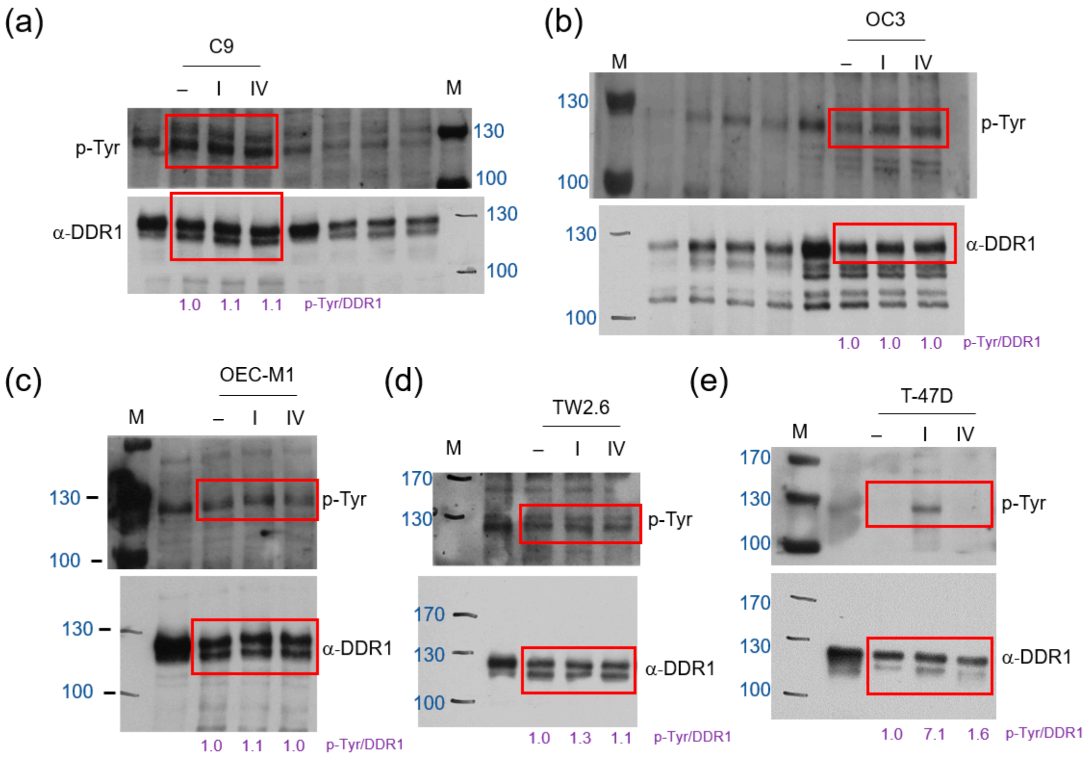

Figure S4. Uncropped scans of Western blots in Figure 3c. In each sample, ratio of phosphorylated DDR1 intensity to that of total DDR1 was quantitated by using ImageJ. The ratios in each untreated group were set to 1.

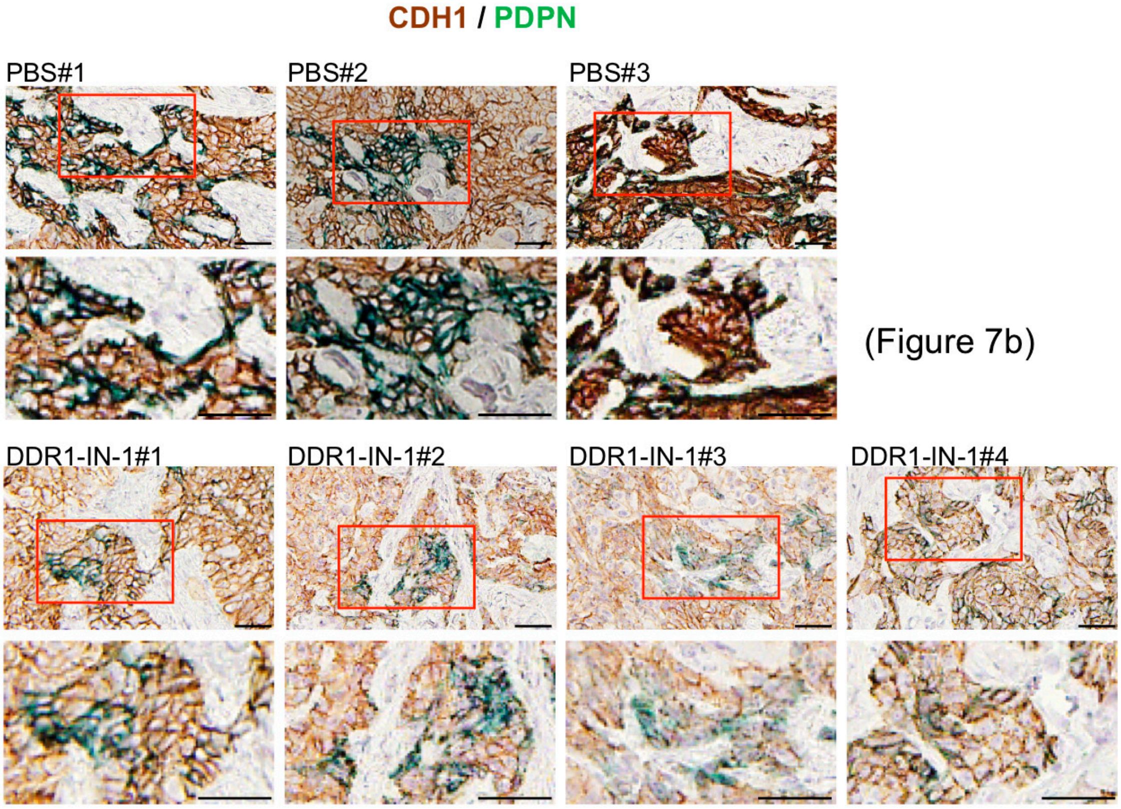

Figure S5. Two magnifications (400× and 800×) of immunohistochemistry images in Figure 7b. Scale bars, 50 μm.

© 2020 by the authors. Licensee MDPI, Basel, Switzerland. This article is an open access article distributed under the terms and conditions of the Creative Commons Attribution (CC BY) license (http://creativecommons.org/licenses/by/4.0/).
